# Disorder-specific effects of polymorphisms at opposing ends of the *Insulin Degrading Enzyme *gene

**DOI:** 10.1186/1471-2350-12-151

**Published:** 2011-11-22

**Authors:** Jasmin Bartl, Claus-Jürgen Scholz, Margareta Hinterberger, Susanne Jungwirth, Ildiko Wichart, Michael K Rainer, Susanne Kneitz, Walter Danielczyk, Karl H Tragl, Peter Fischer, Peter Riederer, Edna Grünblatt

**Affiliations:** 1 Department of Psychiatry, Psychosomatic and Psychotherapy, University Hospital of Wuerzburg, Fuechsleinstr. 15, D-97080 Wuerzburg, Germany; 2 IZKF Laboratory for Microarray Applications, University Hospital of Wuerzburg, Versbacher Str. 7, D-97078 Wuerzburg, Germany; 3 Ludwig Boltzmann Society, L. Boltzmann Institute of Aging Research, Vienna, Austria; 4 Department of Psychiatry, Social Medical Center, Danube Hospital, Langobardenstraße 122, A-1220 Vienna, Austria; 5 Hospital of Child and Adolescent Psychiatry, University of Zurich, Neumuensterallee 9, CH-8032 Zurich, Switzerland

## Abstract

**Background:**

Insulin-degrading enzyme (IDE) is the ubiquitously expressed enzyme responsible for insulin and amyloid beta (Aβ) degradation. *IDE *gene is located on chromosome region 10q23-q25 and exhibits a well-replicated peak of linkage with Type 2 diabetes mellitus (T2DM). Several genetic association studies examined *IDE *gene as a susceptibility gene for Alzheimer's disease (AD), however with controversial results.

**Methods:**

We examined associations of three *IDE *polymorphisms (IDE2, rs4646953; IDE7, rs2251101 and IDE9, rs1887922) with AD, Aβ_42 _plasma level and T2DM risk in the longitudinal Vienna Transdanube Aging (VITA) study cohort.

**Results:**

The upstream polymorphism IDE2 was found to influence AD risk and to trigger the Aβ_42 _plasma level, whereas the downstream polymorphism IDE7 modified the T2DM risk; no associations were found for the intronic variant IDE9.

**Conclusions:**

Based on our SNP and haplotype results, we delineate the model that *IDE* promoter and 3' untranslated region/downstream variation may have different effects on *IDE* expression, presumably a relevant endophenotype with disorder-specific effects on AD and T2DM susceptibility.

## Background

Insulin degrading enzyme (IDE), also known as insulysin, insulin protease or insulinase, is a 110 kDA zinc-dependent metalloprotease of the M16A subfamily, which is coded by a 122 kb spanning, ubiquitously expressed gene on the distal region of the human chromosome 10 q [1]. Apart from its primary target insulin, IDE competitively hydrolyzes multiple proteins including glucagon, atrial natriuretic factor, transforming growth factor-α, and β-endorphin amylin [2,3] These facts suggest a potentially wide role for IDE in the clearance of hormones and bioactive peptides. In addition to its role in insulin catabolism, IDE has been found to degrade β-amyloid (Aβ) 40 and 42 and the amyloid precursor protein (APP) intracellular domain and to eliminate Aβ_42 _neurotoxic effects [4]. The first evidence that IDE might be involved in Aβ_42 _degradation was found by Kurochkin and Goto in 1994, who demonstrated that purified rat IDE efficiently degrades synthetic Aβ_42 _*in vitro *[3]. Subsequently, it was shown that an IDE-like activity from soluble and synaptic membrane fractions of postmortem human brain both degrade Aβ_42 _peptides [5,6]; moreover, IDE doesn't distinguish between endogenous and synthetic Aβ_42 _as substrate *in vitro *[7]

The fact that extracellular Aβ_42 _deposition is one of the hallmarks of the neurodegenerative disorder Alzheimer's disease (AD) made *IDE *a promising candidate involved in disease susceptibility. Several studies associated linkage-peaks over the *IDE *region with late-onset AD (LOAD) [8], age of onset in familial AD [9] and high plasma Aβ_42 _levels [10]. Furthermore, validated linkage peaks with the metabolic syndrome Type 2 Diabetes Mellitus (T2DM) were found in the same region [11,12]. Fakhrai-Rad and colleagues narrowed down the wide range of possible candidate genes to *IDE* via transferring the gene from an inbred rat model of T2DM to a normoglycemic rat, which resulted in the recapitulation of several diabetic features, including hyperinsulinemia and postprandial hyperglycemia [13].

In the present study, we undertook an attempt to associate selected variants of the *IDE *gene with AD, Aβ_42 _plasma levels and T2DM in participants of the Vienna Transdanube Aging (VITA) longitudinal cohort study.

## Methods

### Subjects

Subjects of the present study were from the VITA study which was described previously in greater detail [14,15]. The VITA study investigated the residents of two Viennese districts born between May 1925 and June 1926 (i.e., aged 75 years at inclusion). Data refer to the total cohort at baseline recruitment of 606 individuals who completed physical health check, questionnaires for education, psychosocial activities and neuropsychological examination. The 1^st ^follow-up after 30 months included 468 subjects performing the full health check at the Danube hospital and 30 subjects that were either examined at home or interviewed on the phone. Further 70 subjects refused to attend the follow-up and 38 deceased. At the 2nd follow-up after 60 months 362 subjects attended the full health check at the Danube hospital, whereas 68 subjects were visited at home or only willing to take part in a telephone-interview, thus providing only minimal information. 81 subjects out of the 606 participants deceased between baseline and the 2nd follow-up investigation, 92 subjects refused to take part again and in three cases no contact was possible to establish. A diagnosis of AD was established applying the NINCDS-ADRDA criteria [16]. All participants passed through a consensus conference with regard to the diagnoses of possible or probable AD. The final diagnosis was made by an experienced geronto-psychiatrist. Since the NINCDS-ADRDA diagnosis requires longitudinal information, at baseline all demented subjects were considered as AD positive. In rare cases (n = 16), later (and thus more reliable) examinations revealed "no AD" diagnoses in subjects previously diagnosed as AD positive. For the present analysis, these subjects were treated as AD negative up to the last "no AD" diagnosis. Additional information was obtained in all cases for relevant serum parameters such as cortisol and glucose level and T2DM was diagnosed according to the guide line of the world health organisation. One participant was diagnosed with an untreated schizophrenia; this subject was excluded from AD analyses, however retained in T2DM analyses due to absence of anti-schizophrenic medication, which is known to increase T2DM susceptibility. Additional file 1 - "Demographic information of the VITA study cohort" gives an overview of the subjects examined in this study.

The VITA study was carried out with the permission of the Ethics Committee of the City of Vienna, Austria and each participant gave a written informed consent.

### DNA extraction

DNA was prepared from 2 ml EDTA-blood as previously described [15]. Finally, the DNA was aliquoted into cryo-vials (NUNC, Langenselbold, Germany) and stored frozen at -70 C till requirement.

### IDE SNP selection and genotyping

The most validated *IDE *SNPS were selected after publication research for association studies in AD and T2DM [17-20]. The *IDE *genotypes were determined using TaqMan assay with the real time PCR reaction using specific primers from TaqMan single nucleotide polymorphism (SNP) Genotyping Assay (Applied Biosystems, Darmstadt, Germany), which uses the 5' nuclease assay for amplifying and detecting specific SNP alleles in purified genomic DNA samples. C_22272896_10 (IDE2, rs4646953), C_27104906_10 (IDE7, rs2251101) and C_12116624_10 (IDE9, rs1887922) assays were used for SNP typing. Analysis of the genotypes was conducted on the iCycler software with allelic discrimination program (Bio-Rad, Munich, Germany).

### Aβ_1-42 _plasma level

Plasma levels of amyloid were determined by a double-antibody sandwich enzyme-linked immunosorbent assay method (Innogenetics NV, Ghent, Belgium). The INNOTEST β-amyloid (1-42) allows the specific and reliable measurement of (1-42) amyloid peptides in plasma [21]. The detection range is 5-1000 pg/ml.

### Statistical analysis

Prior to association analysis, *IDE *SNPs were tested for Hardy-Weinberg equilibrium (HWE) with a one degree of freedom χ^2^-test; no significant departures were detected (all p-values > 0.001). Associations in the different time points were tested with logistic regression when the outcome variable was binary (no/yes), or with linear regression when influence on a continuous outcome was examined. For longitudinal association analysis, generalized estimating equations were used. Genotypic associations were performed using three different models: in the additive model, each individual's risk allele count entered the regression; the dominant model considered the presence of at least one risk allele and in the recessive model, a genotypic risk was only present if the individual was homozygous for the risk allele. The Akaike Information Criterion (AIC) was used to choose the best model. In single SNP analysis the polymorphism's minor allele was assumed to be the risk allele; all minor allele frequencies were above our inclusion threshold of 5%. In haplotype analysis, each haplotype allele was tested against all other alleles; to account for phase uncertainty, each allele's posterior probability was incorporated into the model. Haplotypes were defined with the expectation-maximization algorithm [22]. Individuals with any missing values in *IDE *genotypes (n = 38 of 606 in the original VITA cohort) were removed from haplotype analyses. Only common haplotypes with a frequency of at least 5% were analyzed. Associations were considered to be significant at α = 0.05; due to the limited sample size of this study, the reported nominal p-values were not adjusted for multiple testing. Power estimates for the determined effect sizes refer to the described analysis settings and result from the comparison of test statistic distributions under the null and alternative hypothesis. All association and power analyses were performed in R version 2.10.0 using the packages geepack, pwr, powerMediation and SimHap (all obtained from http://www.r-project.org).

## Results

We examined the associations of *IDE *SNPs (IDE2, rs4646953; IDE7, rs2251101; IDE9, rs1887922) and derived haplotypes (on the coding strand in genomic order 5'-IDE2-IDE9-IDE7-3') with AD and Aβ_42 _plasma levels, as well as with T2DM in unrelated subjects participating in the VITA longitudinal cohort study. Further information can be found in Additional file 2 - *"Insulin degrading enzyme *genotype distributions and tests for Hardy-Weinberg equilibrium" and Additional file 3 -"Power calculations for examined markers and outcomes".

### *Insulin degrading enzyme *polymorphisms and Alzheimer's disease

Genetic associations of *IDE *with AD used dominant models. In order to prove the independence of the observed effects from known risk factors like presence of *APOE *ε4 alleles and female sex, respectively, those covariates were included in multivariate regressions. This revealed a significant association of IDE2 (p = 0.03, see Table 1) in subjects that attended the 60 months follow-up (t = 60). The minor allele (C on the coding strand) was found to have a protective effect (OR = 0.55); longitudinally, the effect size did not undergo significant monotonic changes over time (p = 0.41, see Table 1). Results from haplotype analysis reflect the findings of single marker analysis in that the haplotype that carries the IDE2 risk allele together with the major alleles of IDE9 and IDE7 is also associated with AD at t = 60 (see Table 2): haplotype CAA conveys a significantly (p = 0.02) protective ( OR = 0.5) effect. As in single marker analysis, the observed effect size did not undergo significant monotonic changes over time (see Table 2).

**Table 1:** Association results of *insulin degrading enzyme *SNPs

		AD		**Aβ**_**42 **_**plasma level [pg/ml]**		T2DM	
**Polymorphism (major/minor allele)**	**focus**	**OR (95% CI)**	**p-value**	**slope (95% CI)**	**p-value**	**OR (95% CI)**	**p-value**

IDE2 (T/C)	baseline	-	-	22.36 (8.08 - 36.6)	**0.002**	0.82 (0.23 - 2.86)	0.75

IDE2 (T/C)	1^st ^follow-up	0.66 (0.36 - 1.21)	0.18	21.04 (2.08 - 40)	**0.03**	0.96 (0.3 - 3.05)	0.95

IDE2 (T/C)	2^nd ^follow-up	0.55 (0.32 - 0.95)	**0.03**	35.26 (12.8 -57.8)	**0.002**	1.32 (0.41 - 4.24)	0.64

IDE2 (T/C) × examination	longitudinal	1.13 (0.84 - 1.43)	0.41	8.23 (1.93 -14.5)	**0.01**	1.27 (0.81 - 1.73)	0.31

IDE7 (A/G)	baseline	1.74 (0.63 - 4.78)	0.28	-5.24 (-16.7 - 6.2)	0.37	2.4 (1.25 - 4.63)	**0.009**

IDE7 (A/G)	1^st ^follow-up	1.26 (0.77 - 2.07)	0.35	-5.31 (-21 -10.3)	0.51	2.62 (1.32 - 5.18)	**0.006**

IDE7 (A/G)	2^nd ^follow-up	0.97 (0.62 - 1.52)	0.89	-14.27 (-33.7 - 5.2)	0.15	3.47 (1.72 - 6.97)	**0.0005**

IDE7 (A/G) × examination	longitudinal	0.86 (0.56 - 1.16)	0.33	-3.37 (-8.2 - 1.49)	0.18	1.1 (0.91 - 1.3)	0.32

IDE9 (A/G)	baseline	1.87 (0.71 - 4.92)	0.21	-7.36 (-19.5 - 4.8)	0.24	1.65 (0.72 - 3.79)	0.24

IDE9 (A/G)	1^st ^follow-up	1.02 (0.62 - 1.69)	0.94	-8.39 (-24.9 - 8.1)	0.32	1.17 (0.46 - 3.0)	0.75

IDE9 (A/G)	2^nd ^follow-up	0.99 (0.62 - 1.58)	0.96	-11.1 (-31.8 - 9.7)	0.3	1.75 (0.7 - 4.37)	0.23

IDE9 (A/G)× examination	longitudinal	0.88 (0.57 - 1.19)	0.42	-0.85 (-5.5 - 3.83)	0.72	0.98 (0.78- 1.17)	0.81

*Insulin degrading enzyme (IDE) *SNPs (IDE2, rs4646953; IDE7, rs2251101; IDE9, rs1887922) were associated with Alzheimer's disease (AD), amyloid β_42 _(Aβ_42_) plasma levels and Type 2 diabetes mellitus (T2DM). Effect sizes and nominal p-values were derived from multivariate regression analysis with sex and presence of *apolipoprotein E (APOE *ε4) alleles as covariates in regressions on AD affection and Aβ_42 _plasma level, respectively. T2DM affection as outcome used sex and BMI as covariates. Longitudinal analysis examined interactions of all covariates with the examination (baseline, 1^st ^and 2^nd ^follow-up). Associations were calculated using dominant models for AD, additive models for Aβ_42 _plasma level and recessive models for T2DM. Nominally significant p-values (p < 0.05) are shown in **bold**.

**Table 2:** Association results of *insulin degrading enzyme *haplotypes

haplotype				AD		**Aβ**_**42 **_**plasma level [pg/ml]**		T2DM	
**5' IDE2**	**IDE9**	**'3 IDE7**	**focus**	**OR (95% CI)**	**p-value**	**Slope (95% CI)**	**p-value**	**OR (95% CI)**	**p-value**

T	A	A	baseline	2.01 (0.46 -9.47)	0.34	-5.8 (-16.1 - 4.5)	0.27	1.01 (0.63 -1.61)	0.98

T	A	A	1^st ^follow-up	1.96 (0.98 - 3.9)	0.06	-5.33 (-19.5- 8.86)	0.46	0.83 (0.5 - 1.38)	0.46

T	A	A	2^nd ^follow-up	1.51 (0.85 -2.68)	0.16	-7.37 (-24.7 -9.95)	0.41	0.92 (0.54 -1.59)	0.78

T	A × examination	A	longitudinal	0.94 (0.63 -1.41)	0.77	-0.05 (-0.18- 0.07)	0.4	0.95 (0.84 -1.07)	0.41

T	G	G	baseline	2.23 (0.82 -6.1)	0.12	-6.98 (-20.4- 6.47)	0.31	3.25 (1.31 -8.04)	**0.01**

T	G	G	1^st ^follow-up	1.03 (0.61 -1.74)	0.92	-6.78 (-25.1-11.6)	0.47	2.22 (0.81- 6.05)	0.12

T	G	G	2^nd ^follow-up	0.93 (0.56 -1.53)	0.78	-12.06 (-35.3-1.17)	0.31	3.28 (1.2 - 8.99)	**0.02**

T	G × examination	G	longitudinal	0.82 (0.59 -1.14)	0.25	-0.01 (-0.23- 0.21)	0.92	0.99 (0.79 -1.24)	0.93

T	A	G	baseline	0.91 (0.25 -3.26)	0.89	-0.62 (-17.9-16.7)	0.94	6.0 (1.29 -27.7)	**0.02**

T	A	G	1^st ^follow-up	1.22 (0.66 -2.26)	0.52	-0.75 (-25.13-23.6)	0.95	13.0 (1.47-129.33)	**0.02**

T	A	G	2^nd ^follow-up	1.02 (0.57-1.83)	0.94	-13.76 (-43-15.48)	0.36	8.66 (1.54 -48.77)	**0.01**

T	A × examination	G	longitudinal	0,99 (0.69- 1.43)	0.97	0.24 (-0.45- 0.94)	0.49	1.28 (0.64 - 2.6)	0.49

C	A	A	baseline	-	-	26.36 (10.84-41.88)	**0.001**	0.34 (0.04 -2.63)	0.3

C	A	A	1^st ^follow-up	0.65 (0.34-1.23)	0.18	25.93 (5.21-46.64)	**0.01**	0.59 (0.12 -2.82)	0.51

C	A	A	2^nd ^follow-up	0.5 (0.28- 0.88)	**0.02**	41.5 (17 - 66)	**0.001**	0.87 (0.18- 4.14)	0.86

C	A × examination	A	longitudinal	1.08 (0.81-1.46)	0.59	0.23 (-0.38 -0.86)	0.45	1.26 (0.69 -2.32)	0.45

*Insulin degrading enzyme (IDE) *haplotypes (IDE2, rs4646953; IDE7, rs2251101; IDE9, rs1887922) with frequencies > 5% were associated with Alzheimer's disease (AD), amyloid β_42 _(Aβ_42_) plasma levels and Type 2 diabetes mellitus (T2DM). Effect sizes and nominal p-values were derived from multivariate regression analysis with sex and presence of *apolipoprotein E (APOE *ε4) alleles as covariates in regressions on AD affection and Aβ_42 _plasma level, respectively. T2DM affection as outcome used sex and BMI as covariates. Longitudinal analysis examined interactions of all covariates with the examination (baseline, 1^st ^and 2^nd ^follow-up). Associations were calculated using dominant models for AD, additive models for Aβ_42 _plasma level and recessive models for T2DM. Nominally significant p-values (p < 0.05) are shown in **bold**.

### *Insulin degrading enzyme *polymorphisms and amyloid β_42 _plasma level

**Figure 1:**
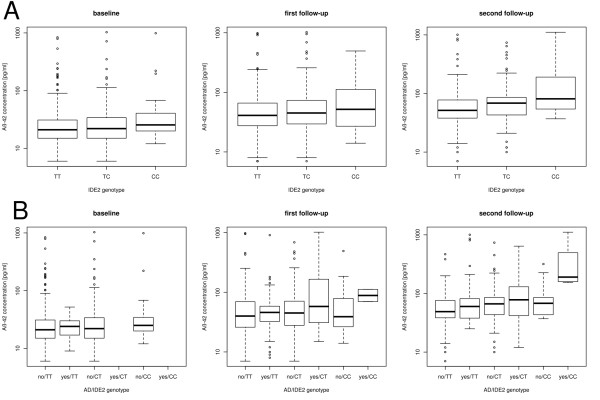
**Amyloid β**_**42 **_**(Aβ**_**42**_) **plasma levels [pg/ml]**. A) in insulin degrading enzyme IDE2 SNP (rs4646953) genotypes and B) in IDE2 genotypes stratified by Alzheimer's disease (AD) status (AD no/yes) at baseline, 1^st ^and 2^nd ^follow-ups.

We then examined the additive influence of *IDE *SNP alleles and haplotypes on the endophenotype Aβ_42 _plasma level. Multivariate regressions included *APOE *ε4 status and sex as covariates. The analyses concordantly revealed that with each minor C allele of IDE2 the Aβ_42 _plasma level increases between 21.04 and 35.26 pg/ml, depending on time point of analysis (see Table 1 and Figure 1A). This finding was significant in all time points (see Table 1). Longitudinal analysis revealed that the observed effect significantly (p = 0.01) increased with each follow-up by 8.23 pg/ml (see Table 1). Of note, an increase in Aβ_42 _plasma level was not exclusive to IDE2 risk allele carriers, but was also observed in individuals homozygous for the major allele (see Table 3 and Figure 1). Since an increased Aβ_42 _plasma level is a known risk factor for AD, we also examined the interaction of the IDE2 genotype with the AD disease status on the Aβ_42 _plasma level. The effect sizes at t = 30 and t = 60 reveal that the IDE2 C allele as well as AD affection both lead to an increased Aβ_42 _plasma level, however that the combined effect is much more pronounced than the sum of both effects (see Table 4 and see Figure 1B). However, the effects for AD and its interaction with the IDE2 genotype are only significant at t = 60, while the effect of IDE2 alone reaches only marginal significance (see Table 4). On multimarker level, we found each CAA haplotype to significantly increase the Aβ_42 _plasma level between 25.93 and 41.5pg/ml, depending on time point of analysis (see table 3B). Longitudinally, we observed no increasing haplotype effect on Aβ_42_ plasma level between examinations (see Table 2).

**Table 3:** Longitudinal analysis of amyloid β_42 _(Aβ_42_) plasma levels in different insulin degrading enzyme 2 (SNP rs4646953) genotypes

IDE2 genotype	**Aβ**_**42 **_**plasma level [pg/ml]**	
	**slope (95% CI)**	**p-value**

all genotypes	23.7 (20.34 - 27.07)	< 2 ∙ 10

only T/T	21.33 (17.73 - 24.94)	< 2 ∙ 10^-16^

only T/C	25.84 (16.52 - 35.17)	5.6 ∙ 10^-8^

only C/C	43.28 (27.7 - 58.86)	5.23 ∙ 10^-8^

Effect sizes and nominal p-values were derived from multivariate regression analysis with sex and presence of *apolipoprotein E (APOE *ε4) alleles as covariates. All IDE2 (SNP rs4646953) genotype groups display a highly significant increase in Aβ_42_plasma concentration between each of the three examinations.

**Table 4:** Interaction analysis of insulin degrading enzyme 2 (SNP rs4646953) genotype

parameter (baseline/risk)	examination	slope (95% CI)	p-value
IDE2 (T/C)	baseline	20.72 (6.23 - 35.21)	**0.005**
AD (no/yes)		-7.45 (-50.34 - 35.45)	0.73
IDE2 × AD		-	-

IDE2 (T/C)	1^st ^follow-up	13.60 (-6.92 - 34.11)	0.19
AD (no/yes)		7.36 (-23.75 - 38.48)	0.64
IDE2 × AD		33.91 (-22.02 - 89.84)	0.24

IDE2 (T/C)	2^nd ^follow-up	21.41 (-3.43 - 46.25)	0.09
AD (no/yes)		36.27 (3.72 - 68.82)	**0.03**
IDE2 × AD		76.89 (24.01 - 129.77)	**0.005**

Analysis of interaction of insulin degrading enzyme SNP rs4646953 *(IDE2) *genotype and Alzheimer's disease (AD) status on amyloid β_42 _(Aβ_42_) plasma level is shown. Effect sizes and nominal p-values were derived from multivariate regression analysis with sex and presence of *apolipoprotein E (APOE *ε4) alleles as covariates. Nominally significant p-values (p < 0.05) are shown in **bold**.

### *Insulin degrading enzyme *polymorphisms and type 2 diabetes mellitus

Genetic associations of *IDE *with T2DM used recessive models. In order to prove the independence of the observed effects from known risk factors like body mass index (BMI) and female sex, those covariates were included in multivariate regressions. In all three time points, we found the minor G allele of IDE7 to bear a significant (p<0.01) genotypic risk to develop T2DM, with an initial (t = 0) OR of 2.4, that continually increased slightly (OR = 1.1) but not significantly (p = 0.32) to 3.47 at t = 60 (see Table 1). Haplotype analyses revealed the association of haplotypes TAG and TGG with T2DM, in case of the first in all three time points, in case of the second only at t = 0 and t = 60 (see Table 2). In line with the single marker result for IDE7, both haplotypes convey a genetic risk to become diabetic with significant effect sizes ranging from 3.25 to 3.28 for TGG and from 6 to even 13 for TAG, depending on time point of analysis (see Table 2). Longitudinally, the associated haplotypes did not exhibit significant monotonic changes in effect sizes (see Table 2).

## Discussion

The identification of a late-onset AD risk locus on chromosome 10q which also affects plasma Aβ_42 _levels, has led to the search for functional candidates in this region [8,10]. Because of its mapping to the AD linkage peak and of the ability of its gene product to degrade Aβ_42_, *IDE *was proposed to harbour a plausible origin for the observed effects. Since *IDE *is also well-known to be associated with increased risk for T2DM [12,23,24], the present study aimed at examining the effects of selected common *IDE *polymorphisms on both disease outcomes as well as on the plasma Aβ_42 _level in subjects participating in the longitudinal VITA cohort study. The analyzed polymorphisms should serve as proxies to partly capture the common allelic variation in the up- (IDE2) and downstream (IDE7) region, as well as in the gene body (IDE9) of *IDE*. Our finding that we were not able to detect associations of IDE9 with neither examined trait leads us to the hypothesis that common *IDE *variants influencing the primary structure of the IDE protein (i.e. non-synonymous SNPs and those affecting splicing junctions, respectively) may have no major impact on AD and T2DM susceptibility. However, despite extended linkage disequilibrium (LD) in the *IDE *region (see Additional file 4 - "Linkage disequilibrium in the *insulin degrading enzyme *region"), a single SNP may not fully represent the allelic variation throughout the gene. In fact, 13 tag SNPs of 45 SNPs genotyped in the HapMap CEU panel (release 24) are needed to capture the variation inside IDE with a mean r^2 ^of 0.99 (data not shown). Therefore the possibility remains that deleterious variants not in LD with IDE9 exist, e.g. rare IDE variants with possibly large effects on our examined traits. While this remains speculative, we found that allelic variation at opposing ends of IDE is associated with different outcomes: the upstream and 5'-untranslated region (UTR) harbours polymorphisms presumably modifying the AD disease risk and the Aβ_42 _plasma level, whereas 3'-UTR and downstream variants may trigger T2DM susceptibility.

There is evidence that the IDE level and therefore Aβ_42 _degrading activity is lower in AD brains than in those of unaffected subjects [6], which can be also an effect of oxidative stress (OS). OS is considered to be a key mechanism in the pathophysiology of AD and is characterized by increased highly reactive oxygen species (ROS) production and decreased antioxidant defence [25,26]. Interestingly the catalytic activity of IDE is reduced through ROS and also the enzymatic activity toward Aβ hydrolysis is decreased [27]. Nonetheless, this has to be further investigated, as it might provide additional link between AD and T2DM. Furthermore, studies on pro-oxidants could be equally important in order to develop new treatment avenues for AD.

A recent study of Zuo and Jia found that variants in the proximal *IDE *promoter increase transcription and thus provide a possible mechanism explaining the protective effect on AD susceptibility found for the examined polymorphisms [28]. Among those, IDE2 overlapped with our study, however with different results: while we found an association with AD in our Vienna-based cohort, IDE2 was not associated in the mentioned Han Chinese sample. This reflects the situation in AlzGene meta-analyses, which list a slightly protective odds ratio (OR) of 0.93 for IDE2 in Central European study populations and an overall OR of 1.0 (i.e. no effect) if Asian studies are included [29]. This makes clear that IDE2 is unlikely to be the causal variant and furthermore raises the possibility that LD between IDE2 and the presumed functional allele varies between Central European and Asian populations. Given this, our present study and that of Zuo and Jia [28] agree that promoter variants leading to increased IDE transcription protect against AD. Correspondingly, one would expect Aβ_42 _plasma levels to be lower in individuals carrying high expression variants, but intriguingly our results show the opposite (see Table 1 and 2). This contradicts the results from a recent study which found high *IDE *expression variants to be correlated with lower Aβ plasma levels, however, Aβ_40 _contributed more to the observation than Aβ_42 _[30]. Plasma Aβ_42 _levels are thus not reliably predicted by *IDE *polymorphisms alone, because also *trans*-acting variants were shown to influence the expression *of IDE *[31]. Furthermore, due to the wide range of IDE substrates, an important determinant for Aβ_42 _degrading activity is the concentration of the primary IDE target (i.e. insulin), illustrated by the finding that even Aβ_40 _clearance is effectively inhibited by insulin [32]. This notion is supported by our observation that a large proportion of the Aβ_42 _plasma level is explained by factors different from the *IDE *promoter genotype (see Table 4).

Hallmarks of T2DM are the presence of insulin resistance and insulin receptor insensitivities. Insulin resistance precedes the onset of T2DM for years [33] and results in compensatory hyperinsulinemia, which is the first step to developing T2DM [34]. Limited capacities to degrade increased plasma insulin levels contribute to development of T2DM. Our association of the downstream variant *IDE7 *with T2DM might be an indirect signal that extends from the IDE 3'-UTR over LD. The presumed risk allele may attenuate translation or reduce the stability of the *IDE *mRNA, thus challenging IDE activity and promoting hyperinsulinemia, explaining the increased T2DM susceptibility.

Based on these assumptions, we delineate the model that polymorphisms at opposing ends of the *IDE *gene may lead to expression changes with consequences on susceptibility to different diseases: promoter variation presumably increases *IDE *expression and protects from AD, while 3'-UTR variation is assumed to decrease *IDE *expression and to increase T2DM risk. Correspondingly, haplotypes that carry both associated alleles on a single DNA molecule (5'-CGG-3' and 5'-CAG-3') should reveal a balanced (i.e. no) effect on AD and T2DM. Due to the low frequencies of these haplotypes (0.5% and 0.2%, data not shown) in the VITA study cohort, we were however not able to reliably examine this balancing effect. Despite the plausibility of the model in the context of the VITA study cohort, validation of the model clearly requires further examinations, including the determination of the IDE activity, which is expected to provide deeper insight into disease causing mechanisms [35].

## Conclusions

Based on our SNP and haplotype results, we delineate the model that *IDE *promoter and 3'-UTR/downstream variation may have opposing effects on *IDE *expression, which is assumed to be a relevant endophenotype with disorder-specific effects on AD and T2DM susceptibility. As a starting point for targeted investigations, the present study provides insight how variation in the *IDE *gene contributes to link the pathophysiologically different diseases AD and T2DM.

## Competing interests

The authors declare that they have no competing interests.

## Authors' contributions

Authors EG and PR designed and managed the study, author JB wrote the first draft of the manuscript, author CJS performed the statistical analysis helped to draft the manuscript, authors SK, PF helped to draft the manuscript and discussed the data, authors MH, SJ, IW, MKR, WD, KHT collected the data and samples of the patients and helped to organized the study. All authors read and approved the final manuscript.

## Pre-publication history

The pre-publication history for this paper can be accessed here:

http://www.biomedcentral.com/1471-2350/12/151/prepub

## Supplementary Material

Additional file 1 **Demographic information of the VITA study cohort**. Abbreviations used: AD = Alzheimer's disease; BMI = body mass index; SD = standard deviation; T2DM = Type 2 diabetes mellitus. Click here for file

Additional file 2 ***Insulin degrading enzyme *genotype distributions and tests for Hardy-Weinberg equilibrium**. *Insulin degrading enzyme (IDE) *genotype distributions and tests for Hardy-Weinberg equilibrium (HWE) in analysis subgroups defined by cross-sectional outcomes. No significant departures from HWE were detected (all p > 0.001). Click here for file

Additional file 3 **Power calculations for examined markers and outcomes**. The presented additional file 3 indicate the power to find nominally significant associations (p < 0.05) given analysis settings used for the estimation of effect sizes shown in Tables 1 and 2. Abbreviations used: Aβ_42 _= amyloid beta 1-42 plasma concentration; AD = Alzheimer's disease; IDE2 = rs4646953; IDE7 = rs2251101; IDE9 = rs1887922; SNP = single nucleotide polymorphism. Click here for file

Additional file 4 **Linkage disequilibrium in the *insulin degrading enzyme *region**. Linkage disequilibrium (LD) in the *IDE *gene region ± 10 kb is shown based on D' values between single nucleotide polymorphisms (SNPs) genotyped in the HapMap CEU panel (release 24). LD colour scheme corresponds to default settings used in Haploview. Positions of SNPs examined in the present study are indicated in blue. Of note, rs4646953 (IDE2) has not been genotyped in the HapMap project, therefore no LD information is available for this SNP. Click here for file
