# Supplementary material for: Disorder-specific effects of polymorphisms at opposing ends of the Insulin Degrading Enzyme gene
Source: BMC Med Genet. 2011 Nov 22;12:151. doi: 10.1186/1471-2350-12-151 (PMC3266204; doi:10.1186/1471-2350-12-151)
Supplement: Additional file 1 — Demographic information of the VITA study cohort. Abbreviations used: AD = Alzheimer's disease; BMI = body mass index; SD = standard deviation; T2DM = Type 2 diabetes mellitus. [file 1471-2350-12-151-S1.PDF]

Additional file 1 – Demographic information of the VITA study cohort.

Abbreviations used: AD = Alzheimer's disease; BMI = body mass index; SD = standard deviation; T2DM = Type 2 diabetes mellitus;

examination	parameter	total sample	with *IDE* genotypes			
			AD		T2DM	
			no	yes	no	yes
baseline	**sex** male / female	247 / 359	232 / 321	5 / 13	194 / 281	44 / 53
	***APOE* ε4** no / yes	469 / 124	429 / 116	13 / 4	365 / 103	78 / 17
	**BMI** mean ± SD	27.1 ± 3.87	27.14 ± 3.81	24.82 ± 3.29	26.93 ± 3.66	27.76 ± 4.42
**1st follow-up**	**sex** male / female	193 / 305	150 / 239	35 / 46	147 / 242	42 / 49
	***APOE* ε4** no / yes	383 / 103	310 / 74	52 / 26	298 / 85	72 / 16
	**BMI** mean ± SD	27.14 ± 3.91	27.32 ± 3.84	26.22 ± 3.95	26.88 ± 3.67	28.18 ± 4.67
**2nd follow-up**	**sex** male / female	163 / 267	107 / 181	48 / 60	124 / 207	35 / 45
	***APOE* ε4** no / yes	327 / 93	229 / 56	73 / 33	249 / 78	65 / 13
	**BMI** mean ± SD	26.75 ± 4.02	27 ± 4.15	25.92 ± 3.55	26.59 ± 3.88	27.14 ± 4.34
