# Supplementary material for: Disorder-specific effects of polymorphisms at opposing ends of the Insulin Degrading Enzyme gene
Source: BMC Med Genet. 2011 Nov 22;12:151. doi: 10.1186/1471-2350-12-151 (PMC3266204; doi:10.1186/1471-2350-12-151)
Supplement: Additional file 2 — Insulin degrading enzyme genotype distributions and tests for Hardy-Weinberg equilibrium. Insulin degrading enzyme (IDE) genotype distributions and tests for Hardy-Weinberg equilibrium (HWE) in analysis subgroups defined by cross-sectional outcomes. No significant departures from HWE were detected (all p > 0.001). [file 1471-2350-12-151-S2.PDF]

Additional file 2 – *Insulin degrading enzyme* genotype distributions and tests for Hardy-Weinberg equilibrium.

Insulin degrading enzyme (*IDE)* genotype distributions and tests for Hardy-Weinberg equilibrium (HWE) in analysis subgroups defined by cross-sectional outcomes. No significant departures from HWE were detected (all $p > 0.001$).

SNP	group	genotypes	distribution [%]	n	p-value
IDE2, rs4646953	AD	TT / TC / CC	81.02 / 16.06 / 2.92	137	0.094
	no AD	TT / TC / CC	76.91 / 19.63 / 3.46	433	0.004
	T2DM	TT / TC / CC	80.87 / 14.78 / 4.35	115	0.008
	no T2DM	TT / TC / CC	76.97 / 19.74 / 3.29	456	0.006
IDE7, rs2251101	AD	AA / AG / GG	45.26 / 46.72 / 8.03	137	0.403
	no AD	AA / AG / GG	48.96 / 41.8 / 9.24	433	0.945
	T2DM	AA / AG / GG	49.12 / 34.21 / 16.67	114	0.019
	no T2DM	AA / AG / GG	47.92 / 45.08 / 7	457	0.093
IDE9, rs1887922	AD	AA / AG / GG	62.04 / 33.58 / 4.38	137	0.877
	no AD	AA / AG / GG	62.27 / 31.25 / 6.48	432	0.07
	T2DM	AA / AG / GG	63.48 / 28.7 / 7.83	115	0.113
	no T2DM	AA / AG / GG	61.98 / 32.53 / 5.49	455	0.401
