# Supplementary material for: Disorder-specific effects of polymorphisms at opposing ends of the Insulin Degrading Enzyme gene
Source: BMC Med Genet. 2011 Nov 22;12:151. doi: 10.1186/1471-2350-12-151 (PMC3266204; doi:10.1186/1471-2350-12-151)
Supplement: Additional file 3 — Power calculations for examined markers and outcomes. The presented additional file 3 indicate the power to find nominally significant associations (p < 0.05) given analysis settings used for the estimation of effect sizes shown in Tables 1 and 2. Abbreviations used: Aβ42 = amyloid beta 1-42 plasma concentration; AD = Alzheimer's disease; IDE2 = rs4646953; IDE7 = rs2251101; IDE9 = rs1887922; SNP = single nucleotide polymorphism. [file 1471-2350-12-151-S3.PDF]

**Additional file 3 – Power calculations for examined markers and outcomes.**

The presented additional file 3 indicate the power to find nominally significant associations ($p<0.05$) given analysis settings used for the estimation of effect sizes shown in Tables 1 and 2.

Abbreviations used: $A\beta_{42}$ = amyloid beta 1-42 plasma concentration; AD = Alzheimer's disease; IDE2 = rs4646953; IDE7 = rs2251101; IDE9 = rs1887922; SNP = single nucleotide polymorphism.

| marker type | marker | focus | power [%] | | |
|---|---|---|---|---|---|
| | | | AD | $A\beta_{42}$ | T2DM |
| SNP | IDE2, C allele | baseline | - | 89.6 | <10 |
| | | 1st follow-up | 24.8 | 72.8 | <10 |
| | | 2nd follow-up | 52.9 | 89.7 | <10 |
| | IDE7, G allele | baseline | 24.6 | 17.9 | 71.3 |
| | | 1st follow-up | 14 | 15 | 74.7 |
| | | 2nd follow-up | <10 | 39.8 | 92.2 |
| | IDE9, G allele | baseline | 19.2 | 26 | 18.1 |
| | | 1st follow-up | <10 | 22.8 | <10 |
| | | 2nd follow-up | <10 | 23.9 | 16.8 |
| haplotype (5'-IDE2-IDE9-IDE7-3') | TAA | baseline | 57 | 19 | <10 |
| | | 1st follow-up | 88 | 13.4 | <10 |
| | | 2nd follow-up | 42.1 | 10.3 | <10 |
| | TGG | baseline | 55.5 | 20.6 | 70 |
| | | 1st follow-up | 86.7 | 16.2 | 25.5 |
| | | 2nd follow-up | 26.5 | 21.8 | 64.5 |
| | TAG | baseline | 64.2 | <10 | 81.6 |
| | | 1st follow-up | 75.7 | <10 | 94 |
| | | 2nd follow-up | 24.2 | 20 | 84.1 |
| | CAA | baseline | - | 93.8 | 16.7 |
| | | 1st follow-up | 46.2 | 82.9 | <10 |
| | | 2nd follow-up | 69.7 | 93.7 | <10 |
