# Supplementary material for: Disorder-specific effects of polymorphisms at opposing ends of the Insulin Degrading Enzyme gene
Source: BMC Med Genet. 2011 Nov 22;12:151. doi: 10.1186/1471-2350-12-151 (PMC3266204; doi:10.1186/1471-2350-12-151)
Supplement: Additional file 4 — Linkage disequilibrium in the insulin degrading enzyme region. Linkage disequilibrium (LD) in the IDE gene region ± 10 kb is shown based on D' values between single nucleotide polymorphisms (SNPs) genotyped in the HapMap CEU panel (release 24). LD colour scheme corresponds to default settings used in Haploview. Positions of SNPs examined in the present study are indicated in blue. Of note, rs4646953 (IDE2) has not been genotyped in the HapMap project, therefore no LD information is available for this SNP. [file 1471-2350-12-151-S4.PDF]

Additional file 4 - Linkage disequilibrium in the *insulin degrading enzyme* region.

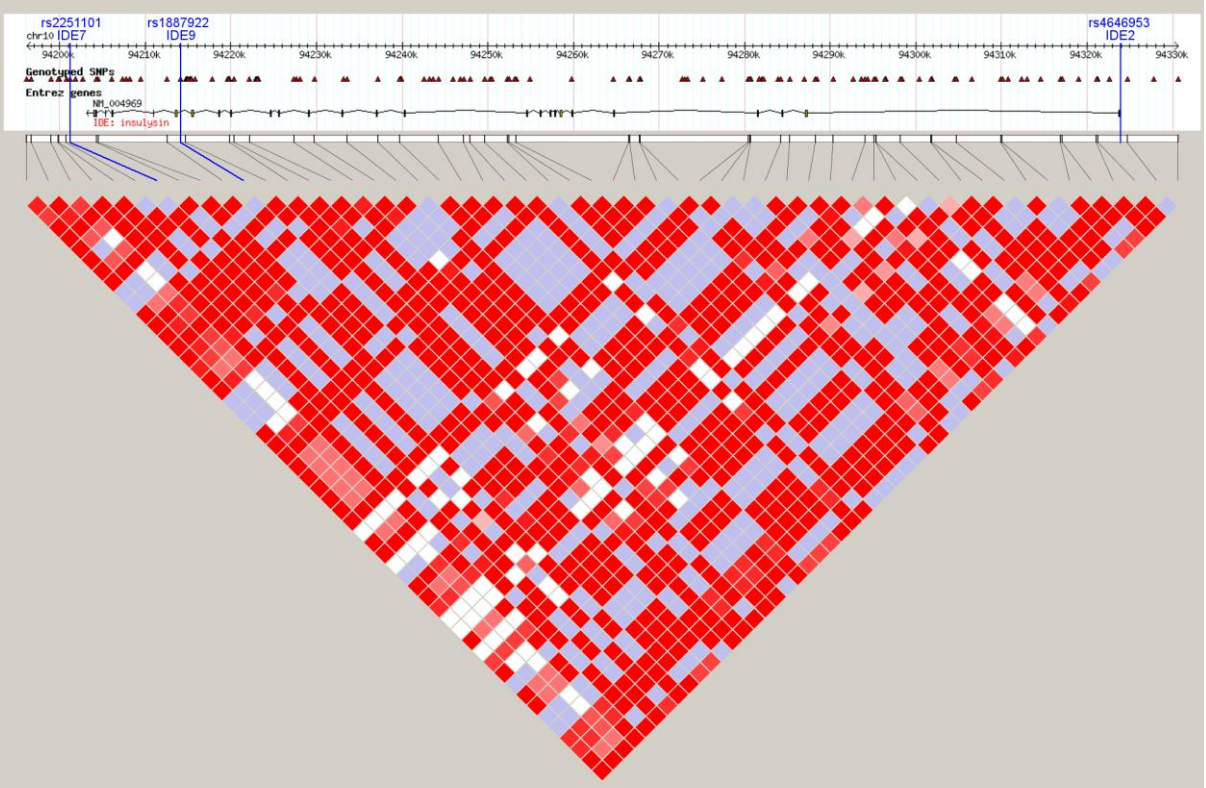

Linkage disequilibrium (LD) in the *IDE* gene region ± 10 kb is shown based on D' values between single nucleotide polymorphisms (SNPs) genotyped in the HapMap CEU panel (release 24). LD colour scheme corresponds to default settings used in Haploview. Positions of SNPs examined in the present study are indicated in blue. Of note, rs4646953 (IDE2) has not been genotyped in the HapMap project, therefore no LD information is available for this SNP.
